# Adaptive Optics Imaging in Diabetic Retinopathy: A Comprehensive Review

**DOI:** 10.22336/rjo.2025.49

**Published:** 2025

**Authors:** Andrada-Elena Mirescu, Dan George Deleanu, George Baltă, Ioana Teodora Tofolean, Florian Baltă, Irina-Elena Cristescu, Sanda Jurja

**Affiliations:** 1 “Ovidius” University of Constanţa, Constanţa, Romania; 2 “Carol Davila” University of Medicine and Pharmacy, Bucharest, Romania; 3 University Emergency Hospital, Bucharest, Romania; 4 Clinical Emergency Eye Hospital, Bucharest, Romania; 5 Retina Clinic, Bucharest, Romania; 6 County Clinical Emergency Hospital of Constanţa, Constanţa, Romania

**Keywords:** adaptive optics, diabetic retinopathy, cones, wall-to-lumen ratio, DR = diabetic retinopathy, NPDR = non-proliferative diabetic retinopathy, PDR = proliferative diabetic retinopathy, SD-OCT = spectral domain optical coherence tomography, FA = fluorescein angiography, AO = adaptive optics, AOSLO = adaptive optics scanning laser ophthalmoscopy, DME = diabetic macular edema, OCTA = optical coherence tomography angiography, DCP = deep capillary plexus, HPi = heterogeneity packing index, RS = retinal sensitivity, IS/ OS = with inner segment/ outer segment, COST = cone outer segment tips, YB = yellow-blue, RG = red-green, TD = vessel diameter, LD = lumen diameter, WT = wall thickness, WLR = wall-to-lumen ratio, WCSA = wall cross-sectional area, MBR = mean blur rate, AIR = arteriolar index ratios, T1D = type 1 diabetes, MAs = microaneurysms, DRIL = disorganization of the retinal inner layers, CFD = computational fluid dynamics

## Abstract

**Objective:**

Diabetic retinopathy (DR), a major diabetes complication, is the fifth leading cause of global blindness and moderate-to-severe visual impairment. DR is categorized into non-proliferative and proliferative forms, with the latter involving neovascularization. Adaptive optics (AO) imaging provides high-resolution, in vivo visualization of retinal microstructures, offering enhanced insight into disease pathology.

**Methods:**

A comprehensive PubMed search was conducted for studies published before August 1, 2025, using the keywords “diabetic retinopathy” and “adaptive optics”. The review focused on studies assessing cone photoreceptors, retinal vasculature, and clinical signs using AO imaging.

**Results:**

Forty-two studies were included. Many examined cone photoreceptor integrity across DR stages, with or without diabetic macular edema (DME). AO imaging revealed microstructural changes, enabling the correlation of cone metrics with other imaging modalities, including optical coherence tomography angiography, electroretinography, microperimetry, and color vision tests. Several studies analyzed capillary-level vascular alterations and their association with DR severity and DME. Some works have integrated AO data with blood flow assessments using techniques such as laser speckle flowgraphy and AO scanning laser ophthalmoscopy. Lastly, several studies have identified clinical signs related to DR and described their features on AO imaging.

**Discussion:**

Adaptive optics imaging reveals early and progressive alterations in photoreceptors, retinal microvasculature, and clinical signs such as microaneurysms and hard exudates in diabetic retinopathy, supporting its role as a valuable tool for understanding disease mechanisms and guiding early diagnosis and monitoring.

**Conclusions:**

Adaptive optics is a valuable imaging tool that improves understanding of retinal microarchitecture in diabetic retinopathy. It aids diagnosis, monitoring, and prognosis by enabling detailed evaluation of cellular and vascular alterations.

## Introduction

Diabetic eye disease remains a growing public health concern, with projections indicating that the global prevalence of diabetes mellitus will rise by 45%, reaching approximately 852 million cases by 2050 [1,2]. With the rising prevalence of diabetes, along with increased life expectancy and an expanding elderly population, the burden of vision loss associated with diabetic retinopathy (DR) is expected to grow substantially in the coming years [3]. Diabetic retinopathy, one of the most common complications of diabetes, currently ranks as the fifth leading cause of blindness and of moderate-to-severe visual impairment worldwide [1,4]. Diabetic retinopathy is a microangiopathy driven by chronic hyperglycemia, leading to pathological alterations in the retinal tissue [1]. While traditionally regarded as a vascular disorder, accumulating evidence indicates that retinal neuronal cells are also compromised in diabetes [5-10]. Neurodegenerative manifestations of diabetic retinopathy include apoptosis of photoreceptors, bipolar cells, and ganglion cells [1].

Diabetic retinopathy is typically divided into two primary forms: non-proliferative diabetic retinopathy (NPDR) and proliferative diabetic retinopathy (PDR), the latter defined by the development of neovascularization [11,12]. Hallmark features of NPDR include microaneurysms, dot-and-blot hemorrhages, hard exudates, and cotton wool spots [12,13]. In contrast, PDR is characterized by neovascularization of the optic disc, iris, or other retinal sites, often accompanied by vitreous hemorrhage or fibrotic changes that may result in tractional retinal detachment [12].

The diagnosis of diabetic retinopathy is initially made through a clinical fundoscopic examination [3]. A growing range of imaging modalities is currently being utilized for the screening, evaluation, diagnosis, and management of diabetic retinopathy. Color fundus photography remains the standard approach for evaluating the severity of diabetic retinopathy in both clinical practice and research settings [1,14]. Imaging modalities such as spectral domain optical coherence tomography (SD-OCT) and fluorescein angiography (FA) are increasingly applied for the screening and management of diabetic retinopathy, as they enable detailed evaluation of retinal abnormalities, including neural layer disorganization, retinal thickening, and vascular leakage, and allow precise characterization of individual DR lesions [1,12,15-20]. Nevertheless, all currently available modalities share a limitation in lateral resolution, which is restricted to about 10–15 μm and therefore insufficient to visualize structures at the cellular scale [1]. Until recently, visualization of the retina at the cellular scale was possible only through histological examination [21]. The introduction of adaptive optics (AO) technology, which corrects wavefront aberrations and achieves an optical resolution of up to 1.4 µm, has transformed the in vivo visualization of ocular structures [21,22].

Adaptive optics was first introduced in astronomy to correct wavefront aberrations caused by atmospheric turbulence, thereby enhancing the sharpness of images of distant celestial bodies [1,21]. In ophthalmology, AO reduces more than 90% of the eye’s optical aberrations, which primarily arise from the tear film, cornea, and lens [1]. Fundamentally, AO serves as a technique for correcting optical aberrations, rather than an independent imaging modality. This technology can be integrated into various ocular imaging systems, including full-field fundus cameras, scanning laser ophthalmoscopes, and optical coherence tomography devices [1,23,24]. Adaptive optics scanning laser ophthalmoscopy (AOSLO) enables non-invasive, in vivo retinal imaging at a microscopic scale, offering detailed visualization of structures such as photoreceptors, nerve fiber layer striations, blood vessels, ganglion cells, and the lamina cribrosa, while also providing dynamic assessment of intravascular blood cell flow down to the capillary level [23-29]. Notably, AOSLO has enabled high-resolution, non-invasive visualization of both neural and vascular alterations in diabetic retinopathy. When combined with complementary modalities, such as SD-OCT, it provides a comprehensive in vivo evaluation of the interplay between neural and vascular pathology in the diabetic retina [1,30,31].

## Materials and methods

A comprehensive literature search was conducted using the PubMed database to identify studies published before August 1, 2025, using the keywords “diabetic retinopathy” and “adaptive optics”. The search was restricted to articles published in English. A total of 90 records were identified between January 1, 2015, and August 1, 2025. After manual screening to remove duplicates, non-human studies, and articles not directly addressing the research focus, 42 studies were selected for further analysis. Exclusion criteria included studies evaluating diabetic patients without diabetic retinopathy, investigations centered solely on adaptive optics imaging acquisition without the inclusion of diabetic cohorts, and research that, although mentioning adaptive optics, primarily focused on other imaging modalities. Studies applying adaptive optics to pathologies unrelated to diabetic retinopathy were also excluded.

## Results

### Cone photoreceptors

Cone photoreceptors were among the first retinal cells to be imaged and quantified using adaptive optics [32,33]. The main parameters evaluated regarding these cells include cone density, spacing, regularity, and reflectivity [34,35]. Cone density is calculated by dividing the number of cones within a region by its area, with age and axial length shown to be important influencing factors [36]. Spacing parameters are derived from the distances between neighboring cones, which are then used to generate a Voronoi diagram of the cone mosaic. This type of analysis also provides information on the uniformity of the mosaic [35,37]. Given that cones are typically organized in a hexagonal lattice, cone regularity is defined by the percentage of cones with exactly six adjacent neighbors [21]. In contrast, reflectivity is measured as the average pixel intensity associated with the cone photoreceptors [21,32].

Several studies have focused on evaluating cone photoreceptors in diabetic patients with various forms of diabetic retinopathy, including those with or without diabetic macular edema, using adaptive optics to assess retinal microstructural alterations. A part of these studies have examined correlations between adaptive optics-derived cone parameters and other imaging modalities, including optical coherence tomography angiography, electroretinography, microperimetry, and color vision assessments. A summary of these studies is provided in **Table 1**.

**Table 1:** Studies investigating cone photoreceptors in DR patients using adaptive optics imaging

Year	Study	Population included	Results
2016	Lombardo M et al. [38]	16 patients with type 1 diabetes mellitus – 8 no DR, eight mild NPDR, and 20 controls	Cone density, linear dispersion index, and heterogeneity packing index were significantly altered in eyes with no DR or NPDR compared to controls, with differences increasing with diabetes duration. Individually, these metrics could not fully distinguish between no DR and controls; however, when combined in a logistic regression model, they achieved 100% accuracy. Thus, adaptive optics biomarkers can reliably detect parafoveal cone abnormalities in type 1 diabetes even before clinical signs of DR appear [38].
2016	Soliman MK et al. [39]	25 patients (29 eyes) with type II diabetes mellitus – no DR and with DR (mild, moderate, severe NPDR, and PDR), and 10 healthy participants (20 eyes)	Cone density was significantly reduced in moderate NPDR and severe NPDR, and PDR, compared with controls, no DR, and mild NPDR. However, no association was observed between cone density and either HbA1c levels or diabetes duration. AO imaging suggests photoreceptor loss increases with DR severity in type II diabetes, being more pronounced in advanced stages due to a greater risk of macular edema and its consequences [39].
2016	Lammer J et al. [40]	53 subjects - (11 no DR, 10 mild NPDR, 11 moderate NPDR, 6 severe NPDR, 5 PDR), and the rest of them were controls	Cone density and spacing were not significantly associated with diabetes, HbA1c, or DR severity. In contrast, decreased cone regularity was related to the presence of diabetes, advancing DR severity, and diabetic macular edema. While density and spacing appear largely preserved in diabetes, altered cone regularity is consistently linked to disease status and progression, highlighting the value of AOSLO assessment of cone arrangement as a potential marker of structural or functional impairment [40].
2017	Nesper PL et al. [41]	11 eyes from 11 diabetic patients -with diabetic retinopathy, ranging from minimal NPDR to high risk and quiescent PDR.	Correlation between SD-OCT, optical coherence tomography angiography (OCTA), and AOSLO imaging: all eight eyes with deep capillary plexus (DCP) non-perfusion showed photoreceptor abnormalities, with six also having outer retinal changes on SD-OCT, while 3 DR eyes with intact DCP perfusion had normal photoreceptors. Compared to DR eyes without non-flow, those with DCP non-flow had a lower heterogeneity packing index (HPi) and parafoveal DCP vessel density, with cone HPi strongly correlating with vessel density. These findings highlight the DCP’s role in photoreceptor oxygenation in diabetic macular ischemia and the sensitivity of AOSLO in detecting subtle changes that may not be apparent on SD-OCT [41].
2017	Mariotti L et al. [42]	6 patients with mild NDPR and two healthy subjects	Cone intensity histograms, mosaic texture metrics (sharpness, entropy), and novel indices (cone/intercone intensity and variogram slope) were evaluated at four retinal sites (2° from the fovea). Histogram distributions were similar across groups, but cone/intercone intensity, variogram slope, and entropy were significantly altered in NPDR. Most measures were stable over time, except the mean cone intensity. These results show disrupted cone reflectance in NPDR and introduce the variogram as a tool to quantify short-range spatial differences [42].
2019	Zaleska-Żmijewska A et al. [43]	36 patients with NPDR (22 mild and 14 moderate) and 20 healthy volunteers	Cone density at 2° eccentricity was significantly reduced in the DR group compared with controls. Both cone density and cone regularity declined progressively with greater DR severity [43].
2020	Ro-Mase T et al. [44]	4 patients no DR, 12 patients with NPDR, 10 patients with PDR, and 13 controls	Correlation between AO, OCTA, and microperimetry: reduced foveal cone density in PDR versus no DR. Parafoveal heterogeneity packing index (HPi) was significantly lower in DR (NPDR and PDR) than in no DR, with no difference between NPDR and PDR. In contrast, foveal HPi showed no group differences. HPi and flow deficits were not significantly correlated in either foveal or parafoveal regions. Still, foveal and parafoveal flow deficits were significantly associated with reduced retinal sensitivity (RS) in NPDR and PDR [44].
2021	Datlinger F et al. [45]	5 eyes of 4 patients (2 eyes PDR, three eyes moderate NPDR)	Correlation between AO-OCT and microperimetry: AO-OCT showed pronounced cone photoreceptor disruption in capillary nonperfusion areas, with inner segment/ outer segment (IS/OS) and cone outer segment tips (COST) abnormalities in 84% and 87% of affected regions versus 9% and 8% in perfused areas. Photoreceptor signal density decreased by 38% at the IS/OS and 39% at the COST, and retinal sensitivity was significantly lower in regions of deep capillary plexus nonperfusion [45].
2023	Viggiano P et al. [46]	40 patients with type 1 diabetes (12 no DR and 28 NPDR) and 10 healthy controls	Correlation between optical coherence tomography angiography and adaptive optics: in the NPDR group, cone metrics were strongly linked to choriocapillaris flow deficits. An increased flow deficit percentage was associated with a higher linear dispersion index, while greater flow deficits corresponded to reduced cone density and a lower heterogeneity packing index. Moreover, cone density exhibited significant negative correlations with perfusion density in both the superficial and deep capillary plexus [46].
2023	Fragiotta S et al. [47]	22 patients with type 1 diabetes and mild NPDR	Correlation between optical coherence tomography angiography and adaptive optics, over the 4-year follow-up: cone density remained stable over time, while cone spacing and arrangement showed significant changes in specific parafoveal sectors. These alterations were linked to progressive perfusion loss in the superficial capillary plexus and choriocapillaris, suggesting that declining vascular support contributes to photoreceptor structural rearrangement [47].
2023	Kupis M et al. [48]	50 diabetic individuals (type 1 or type 2 - moderate NPDR), and 18 healthy volunteers	Over a two-year follow-up, patients with DR had significantly lower cone density in all quadrants at both baseline and follow-up, with a greater decline compared to the control group. Interphotoreceptor spacing was higher in DR and increased over time, remaining significantly different except in the inferior quadrant. Cone regularity was reduced in the DR at baseline and declined further, with significant differences noted in the temporal, nasal, and superior quadrants. The proportion of hexagonal cones decreased in all quadrants in DR, but only in the temporal quadrant of controls [48].
2024	Balas M et al. [49]	48 participants (87 eyes) for photoreceptor data and 36 participants (62 eyes) for vascular data; grouped by: control, mild NPDR, moderate/severe NPDR, and PDR	Photoreceptor parameters differed significantly between DR groups at 2° and 4° eccentricity. In multivariable analysis, cone density and dispersion emerged as the strongest predictors of DR severity. Additionally, all photoreceptor measures showed significant correlations with visual acuity, both overall and across most DR severity groups [49].
2024	Gu Q et al. [50]	255 eyes of 134 diabetes mellitus patients (exclusion criteria - PDR)	No significant differences were found in cone parameters, such as regularity, spacing, dispersion, and hexagonal cone ratio, between non-DME and early DME eyes [50].
2024	Lai C et al. [51]	81 participants (23 healthy controls, 23 preclinical DR, 13 NPDR, 22 PDR)	Correlation between electroretinography, ultra-widefield swept-source optical coherence tomography angiography, and adaptive optics: in preclinical DR, reduced response amplitude was observed without structural or photoreceptor changes. In NPDR, photoreceptor impairment, delayed implicit time, retinal thickening, and reduced deep capillary plexus perfusion were found. In PDR, both delayed implicit time and reduced amplitudes were observed, along with neurovascular impairments. Functional deficits correlated significantly with structural changes across all groups [51].
2024	Vaughan M et al. [52]	26 DM patients (all stages of DR) and 25 healthy controls	Correlation between color vision and cone metrics: in diabetic patients, cone density was significantly reduced at 1° eccentricity compared to controls, while intercellular regularity was also significantly lower at both 1° and 2° eccentricities. An inverse correlation was observed between cone density and yellow-blue (YB) color vision thresholds—indicating that lower cone density was associated with worse YB sensitivity. No significant correlation was found between cone metrics and red-green (RG) thresholds, nor in healthy control participants [52].
2025	Balas M et al. [53]	29 participants (46 eyes) (control, mild NPDR, moderate/severe NPDR, and PDR)	Higher cone density was inversely correlated with both total vessel diameter and lumen diameter. In contrast, greater cone dispersion was associated with larger total vessel diameter and lumen diameter. These relationships were mainly observed in cases of mild NPDR, with no significant correlations detected in more advanced stages [53].
2025	Mirescu AE et al. [54]	4 adults (healthy volunteer, NPDR, PDR, macular telangiectasia type 2)	Cone density was lower in all quadrants of the PDR patient compared to the healthy volunteer. Furthermore, cone density showed a progressive decline with increasing severity of diabetic retinopathy [54].

### Vascular parameters

Adaptive optics can also be used to evaluate vascular parameters, including vascular morphology through measurements of total vessel diameter (TD), lumen diameter (LD), and wall thickness (WT). From these, derived indices such as the wall-to-lumen ratio (WLR), defined as WT divided by LD, and the wall cross-sectional area (WCSA), representing the relationship between LD and TD, can be calculated [21].

Several studies have evaluated vascular parameters in diabetic patients at different stages of retinopathy, both with and without diabetic macular edema, using adaptive optics imaging to assess capillary-level microvascular alterations. In addition, some studies have correlated adaptive optics findings with blood flow velocity measurements obtained through laser speckle flowgraphy, adaptive optics scanning laser ophthalmoscopy, or other imaging techniques. A summary of these studies is presented in **Table 2**.

**Table 2:** Studies investigating vascular parameters in DR patients using adaptive optics imaging

Year	Study	Population included	Results
2017	Luo T et al. [55]	17 NPDR and 26 healthy patients	This study used AOSLO to evaluate retinal vascular branching in NPDR. In healthy eyes, branching exponents for larger vessels were below Murray’s predicted value of 3, whereas NPDR arteries were closer to it (3.09). Small-vessel exponents differed significantly between individuals with diabetes and those without. Bifurcation angles were similar (~78–79°), with the pumping power model providing a better match to the observed angles. NPDR thus alters parent-daughter vessel diameter relationships but not branching angles [55].
2019	Zaleska-Żmijewska A et al. [43]	36 patients with NPDR (22 mild and 14 moderate) and 20 healthy volunteers	Arterial walls were significantly thicker in the DR group, with both the wall-to-lumen ratio and the wall cross-sectional area markedly higher compared to controls [43].
2019	Palochak CMA et al. [56]	39 eyes of 30 patients with diabetes with mild NPDR or no DR, and 21 eyes of 17 healthy controls	Correlation between AOSLO and OCTA: retinal blood velocity was elevated in eyes with no DR and reduced in NPDR across all vessel sizes compared with controls. Blood flow was similarly higher in no DR and lower in NPDR for vessels with a diameter of ≤ 60 μm. Outlier analysis revealed distinct differences in vessel density between low-flow no DR eyes and high-flow NPDR eyes. Overall, blood velocity and flow increase in no DR but decrease in mild NPDR relative to healthy controls [56].
2021	Ueno Y et al. [57]	7 no DR eyes, 36 mild or moderate NPDR eyes, 22 severe NPDR eyes, 32 PDR eyes, and 24 control eyes	Correlation between adaptive optics imaging and laser speckle flowgraphy measurements: AO imaging showed a significantly higher WLR in the PDR group, despite similar external vessel diameters. Mean blur rate (MBR)-vessel was lower in PDR, and WLR correlated negatively with MBR-vessel and positively with disease duration, diabetes stage, blood pressure, and hypertension. Regression analysis identified MBR-vessel, hypertension, and LDL as independent predictors of WLR. Increased wall thickness in PDR caused lumen narrowing and reduced blood flow [57].
2022	Baltă F et al. [58]	57 diabetic patients (19 no DR, 17 NPDR, 21 PDR) and 17 healthy volunteers	Vessel and lumen diameters, as well as wall cross-sectional area, did not differ significantly between groups. Still, the wall-to-lumen ratio was higher in eyes with no DR and PDR compared with controls, remaining significant after adjusting for age and diabetes duration. In people with diabetes, worse visual acuity correlated with greater wall thickness and WLR. Wall thickness and wall cross-sectional area were also significantly higher in eyes with diabetic maculopathy [58].
2023	Kupis M et al. [48]	50 diabetic individuals (type 1 or type 2 - moderate NPDR), and 18 healthy volunteers	At baseline, the lumen and total diameter of the artery did not differ between groups. Still, the DR group exhibited thicker arterial walls and significantly higher WLR and WCSA values compared to the controls. Over a two-year period, the DR group showed increases in wall thickness, WLR, and WCSA, although only the thickening of walls one and two reached statistical significance. In controls, arterial wall thickness, WLR, and WCSA also increased, but final WLR values remained within the normal range [48].
2023	Sapoznik KA et al. [59]	48 participants (26 with diabetes mellitus – no DR, NPDR, PDR) and 22 healthy controls	In diabetic patients, arteriole segments showed significant differences in wall-to-lumen ratio, wall thickness, and arteriolar index ratios (AIR). The individual AIR was also markedly altered in the diabetes group, and subgroup analysis indicated that the presence of DR had a significant impact on AIR [59].
2024	Balas M et al. [49]	48 patients (87 eyes) for photoreceptor data and 36 patients (62 eyes) for vascular data; grouped by: control, mild NPDR, moderate NPDR, severe NPDR, and PDR	Of the vascular parameters assessed, only the wall-to-lumen ratio showed a significant difference between DR groups, and this was confined to 2° eccentricity. All other vascular measures were similar across DR severity groups and did not serve as significant predictors of disease severity [49].
2024	Huang BB et al. [60]	15 no DR 8 with DME and either NPDR or PDR, and 17 healthy eyes	Diabetic patients without DR and those with DME showed significantly higher WLR than healthy controls. In multivariable analysis limited to healthy and DM no DR groups, hypertension was the strongest predictor of WLR, with no significant effect from age or diabetes. However, when all three groups were considered together, diabetes emerged as the primary factor associated with WLR, while age and hypertension were not found to be significantly related [60].
2024	Gu Q et al. [50]	255 eyes of 134 diabetes mellitus patients (exclusion criteria - PDR)	In the upper retina, early DME eyes showed significantly reduced vessel diameter, wall thickness, wall-to-lumen ratio, and vascular wall cross-sectional area compared to non-DME eyes. These findings suggest that early DME may involve localized vasospasm or vasoconstriction [50].
2024	Sampani K et al. [61]	19 patients with type 1 diabetes (no DR, mild-moderate NPDR, severe NPDR-PDR), and five healthy volunteers	Eyes with type 1 diabetes (T1D) showed a significant increase in mean wall thickness compared to controls, with further thickening observed as DR severity progressed. The WLR was also significantly higher in T1D eyes and in those with more advanced stages of DR. However, arteriolar diameters did not differ significantly based on T1D status or DR severity [61].
2025	Mirescu AE et al. [54]	4 adults (healthy volunteer, NPDR, PDR, macular telangiectasia type 2)	The PDR patient exhibited a higher WLR compared to the healthy volunteer. Moreover, a positive correlation was observed between WLR and diabetic retinopathy severity, with patients with PDR exhibiting a greater WLR than those with NPDR [54].
2025	Mirescu AE et al. [62]	69 adults across four groups (control, no DR, NPDR, PDR)	Mean arterial wall thickness and WLR increased progressively from no DR to PDR, with statistically significant elevations in the PDR group compared to controls. Although lumen diameter and total vessel diameter tended to decrease in PDR, these changes were not statistically significant [62].

### Clinical signs

Adaptive optics imaging enables more precise detection of microaneurysms, IRMAs, and neovascularization than fundus photography. Microaneurysms and hemorrhages appear as hyporeflective dots, edema causes image blurring, cystoid spaces show sharp borders, and hard exudates display dark edges [63]. A summary of studies evaluating clinical signs using adaptive optics is presented in **Table 3**.

**Table 3:** Studies investigating clinical signs in DR patients using adaptive optics imaging

Year	Study	Population included	Results
2024	Torm MEW et al. [64]	21 eyes of 11 patients with mild to moderate NPDR and 13 eyes of 10 healthy subjects	In AO-SLO imaging of NPDR eyes, capillary looping, inflections, and dilations were detected in very mild and mild NPDR cases. At the same time, microaneurysms with hyperreflective granular elements appeared in mild to moderate NPDR. Most abnormalities were perfused on OCTA, though some loops showed occlusion or undetectable flow, suggesting hypoperfusion. In one moderate NPDR case, ghost vessels (non-perfused capillaries) were revealed by aligning AO-OCT and AO-OCTA. Combining advanced non-invasive imaging modalities enables earlier and more detailed detection of microscopic DR changes compared to conventional fundus imaging [64].
2019	Cristescu IE et al. [65]	7 patients with diabetes mellitus and diabetic retinopathy	Red lesions on fundus photos appeared hyporeflective on AO, while OCTA differentiated microaneurysms from hemorrhages. Hard exudates exhibited a granular pattern, retinal edema resulted in image blurring, and cystic spaces displayed hyporeflective borders. Thus, AO imaging provides detailed documentation of retinal lesions and holds promise for early DR diagnosis and pathophysiological insight [65].
2018	Hafner J et al. [66]	7 eyes of 5 patients with diabetes mellitus and diabetic retinopathy	In a longitudinal study using AO-OCT, 18 microaneurysms (MAs) in seven eyes of five diabetic patients were monitored over an 18-month period. While all MAs maintained a stable saccular shape in en face view, AO-OCT revealed dynamic changes, including growth, involution, disappearance, and division. Intraluminal hyperreflective material was standard, showing variable patterns of stability, increase, regression, or fluctuation, with some MAs developing new reflectivities over time. AO-OCT thus demonstrated the heterogeneous and evolving structural behavior of MAs in vivo [66].
2018	Lammer J et al. [30]	30 eyes of 29 diabetic patients with DR (3 mild NPDR, eight moderate NPDR, seven severe NPDR, 11 PDR)	One hundred nine microaneurysms imaged with AO-SLO and SD-OCT, wall hyperreflectivity correlated with larger MAs size and adjacent disorganization of the retinal inner layers (DRIL). In contrast, intraluminal hyperreflectivity was linked to perfusion and photographic visibility. Larger MAs were also associated with partial perfusion and ring signs. DRIL correlated with reduced visual acuity. These findings highlight the interplay between vascular and neural pathology in diabetic eyes and suggest that MAs structure and DRIL may serve as markers of functional decline [30].
2018	Loganadane et al. [67]	5 eyes of 3 patients with diabetic retinopathy	In a prospective 8-week study of five eyes with diabetic maculopathy, adaptive optics imaging revealed that dynamic short-term changes in hard exudates are not detectable clinically. Two eyes with resolving macular edema showed dislocation and fragmentation of exudates into smaller foci (resorption exudates), while three eyes with persistent edema exhibited aggregation of foci into larger deposits. These findings demonstrate that adaptive optics can precisely document the evolution of subtle exudates, offering insights into disease mechanisms and potential treatment monitoring [67].
2018	Bernabeu MO et al. [68]	13 eyes of 11 patients with diabetes mellitus and diabetic retinopathy	This study combined AO-SLO with computational fluid dynamics (CFD) to model perfusion in diabetic retinal microaneurysms (MAs). 20 MAs from were analyzed, and four novel indices were proposed: two structural (asymmetry ratio, body-to-neck ratio) and two hemodynamic (shear rate mean drop, wall shear stress mean drop). Results showed that saccular MAs were smaller and more prone to clot formation than fusiform ones, with clots occurring in areas of low shear rate. The body-to-neck ratio strongly correlated with abnormal perfusion parameters. These findings suggest that morphology and CFD-derived indices can help predict the risks of leakage or thrombosis, offering potential biomarkers for the progression of diabetic retinopathy [68].
2019	Karst SG et al. [69]	15 eyes of 10 patients with diabetes mellitus and diabetic retinopathy	Fifty-three retinal microaneurysms in 15 eyes of 10 diabetic patients were imaged with AO-OCT and compared with standard imaging methods. AO-OCT identified feeding or draining vessels in most cases and localized MAs in the inner nuclear layer, connected to the intermediate and deep capillary plexus. OCTA detected only about two-thirds of MAs, while intraluminal hyperreflectivity seen with AO fundus imaging often originated from vessel wall reflections rather than true clots. Overall, AO-OCT provided the most detailed 3D morphological characterization of retinal MAs in vivo [69].

## Discussion

The studies included in this review demonstrate that adaptive optics (AO) imaging can detect early and progressive alterations in both photoreceptors and retinal microvasculature in diabetic retinopathy. Cone density, spacing, and regularity were shown to decline with increasing disease severity, while vascular parameters such as wall thickness and wall-to-lumen ratio reflected microvascular remodeling. AO also enabled the detailed visualization of clinical signs, including microaneurysms, hard exudates, and others, capturing structural dynamics that are not always evident with conventional imaging. Although small cohorts and methodological variability limit current evidence, these consistent findings across cellular, vascular, and lesion-based assessments support AO as a valuable tool for understanding disease mechanisms and potentially guiding early diagnosis and monitoring in diabetic retinopathy.

## Conclusions

In conclusion, adaptive optics imaging is a state-of-the-art technique that enables high-resolution visualization of retinal photoreceptors, microvasculature, and clinical features associated with diabetic retinopathy. By revealing subtle microstructural changes at a cellular level, AO enhances our understanding of DR pathology, particularly its vascular and neurodegenerative components.

This advanced imaging method supports earlier and more precise diagnosis, enables better monitoring of disease progression, and offers valuable insights for treatment evaluation. As AO continues to evolve, it holds strong potential to become a key tool in both clinical practice and research for retinal disorders.
